# Retracted: A Posterior TAP Block Provides More Effective Analgesia Than a Lateral TAP Block in Patients Undergoing Laparoscopic Gynecologic Surgery: A Retrospective Study

**DOI:** 10.1155/2022/9768905

**Published:** 2022-08-20

**Authors:** Anesthesiology Research and Practice

*Anesthesiology Research and Practice* has retracted the article titled “A Posterior TAP Block Provides More Effective Analgesia Than a Lateral TAP Block in Patients Undergoing Laparoscopic Gynecologic Surgery: A Retrospective Study” [1]. Since publication, the authors notified us that the data presented in the article had been found to be unreliable due to suspected misconduct following an investigation by Showa University.

The authors and the editorial board approved this retraction.
